# BAF45b Is Required for Efficient Zika Virus Infection of HAP1 Cells

**DOI:** 10.3390/v13102007

**Published:** 2021-10-06

**Authors:** B. David Persson, Stefan Nord, Richard Lindqvist, Katarina Danskog, Anna K. Överby, Alain Kohl, Hugh J. Willison, Annasara Lenman, Niklas Arnberg

**Affiliations:** 1 Department of Clinical Microbiology, Umeå University, 901 85 Umeå, Sweden; stefan.nord@foi.se (S.N.); richard.lindqvist@umu.se (R.L.); katarina.danskog@umu.se (K.D.); anna.overby@umu.se (A.K.Ö.); anna-sara.lenman@umu.se (A.L.); 2 Laboratory for Molecular Infection Medicine Sweden (MIMS), within the EMBL Nordic Partnership for Molecular Medicine, Umeå University, 901 85 Umeå, Sweden; 3 MRC-University of Glasgow Centre for Virus Research, Glasgow G61 1QH, UK; alain.kohl@glasgow.ac.uk; 4 Institute of Infection, Immunity and Inflammation, University of Glasgow, Glasgow G12 8TA, UK; hugh.willison@glasgow.ac.uk

**Keywords:** Zika virus, flavivirus, BAF45b, DPF1

## Abstract

The 2016 Zika virus (ZIKV) epidemic illustrates the impact of flaviviruses as emerging human pathogens. For unknown reasons, ZIKV replicates more efficiently in neural progenitor cells (NPCs) than in postmitotic neurons. Here, we identified host factors used by ZIKV using the NCI-60 library of cell lines and COMPARE analysis, and cross-analyzed this library with two other libraries of host factors with importance for ZIKV infection. We identified BAF45b, a subunit of the BAF (Brg1/Brm-associated factors) protein complexes that regulate differentiation of NPCs to post-mitotic neurons. ZIKV (and other flaviviruses) infected HAP1 cells deficient in expression of BAF45b and other BAF subunits less efficiently than wildtype (WT) HAP1 cells. We concluded that subunits of the BAF complex are important for infection of ZIKV and other flavivirus. Given their function in cell and tissue differentiation, such regulators may be important determinants of tropism and pathogenesis of arthropod-borne flaviviruses.

## 1. Introduction

Starting in 2015, an increasing number of cases of microcephaly and other neurodevelopmental disorders in newborns [1,2] referred to as congenital Zika syndrome (CZS), along with a wide range of other post-natal neurological disorders [3], were reported from Brazil. In early 2016, the WHO declared the Zika virus (ZIKV) outbreak to a be a public health emergency of international concern [4], and by the middle of 2017, Brazil’s Ministry of Health reported a total of 14,558 suspected cases of microcephaly and other congenital malformations of the central nervous system [5]. ZIKV is transmitted primarily by mosquitos [6] and causes a primary, local infection, but eventually spreads into the central nervous system (CNS) [7] and targets cells of neuronal origin, and, in particular, neural progenitor cells (NPCs) [8], rather than post-mitotic neurons. This infection may lead to hypoplasia of various structures leading to a smaller brain weight, large irregular clusters of immature cells, and Wallerian degeneration [9]. In pregnant mice, ZIKV infection leads to disrupted NPC development and microcephaly, which is associated with altered transcription in neurodevelopment pathways [10,11]. The ZIKV genome is a ~10.8 kb non-segmented, positive-sense single-stranded RNA that is capped at the 5′ end, and lacks a 3′ polyA tail [12]. The genome translates into a single polyprotein that reflects the generic arthropod-born flavivirus genome structure with structural and non-structural (NS) proteins, including the RNA dependent RNA polymerase NS5 and the helicase NS3.

Recent studies have pointed out the importance of microRNA (miRNA), including miR-9, during ZIKV infection, leading to apoptosis-associated microcephaly, reduced numbers of NPCs, and newly formed neurons in embryonic mouse cortex [13,14,15,16]. ZIKV infection in neuronal stem cells also upregulates miR-124-3p, which has been predicted to be involved in microcephaly [17]. Both miR-9 and miR-124 are selectively expressed in mature, post-mitotic neurons and are key regulators of a 12 subunit-containing chromatin-remodeling complex termed BAF (Brg1/brm-associated factor), which is specifically involved in NPC transition from multipotent to post-mitotic neurons [18]. The genes encoding subunits of the BAF complex are associated with mammalian neural development and neurodegenerative disorders including microcephaly and ZIKV disease [13,18,19,20,21,22,23,24]. In NPCs, the complex is termed npBAF (neural-progenitor BAF) and contains four unique factors: BAF45a, BAF45d, BAF53a, and SS18. In post-mitotic neurons, these factors are replaced with BAF45b, BAF45c, BAF53b, and SS18L1, respectively, and the complex is then termed nBAF (neural-specific BAF) (Figure 2A). BAF53a is repressed by the presence of sequences in the 3′ untranslated region that corresponds to the recognition sites in the 3′ region of miR-9 (termed miR-9*) and miR-124 [18]. Mutations in the genes encoding these miRNA result in persistent expression of BAF53a and defective activity-dependent dendritic outgrowth in neurons; overexpression of miR-9* and miR-124 in neural progenitors causes reduced proliferation. ZIKV infection is further associated with the subunits of the BAF complex, in that BAF53a is known to control a transcriptional program that promotes proliferation and suppresses differentiation, in part, through activation of the Hippo-YAP pathway [25], which controls organ size through progenitor cell proliferation and differentiation. This pathway is dysregulated by ZIKV infection [26] and associated with a ZIKV infection-dependent alteration of DNA methylation status of genes involved in the Hippo signaling pathway in NPCs [27].

Several different screening approaches have been used to identify host factors critical for ZIKV infection [16,17,28,29]. In this study, we combined the results from three screens—two published by others, and one performed in our laboratory—to identify novel determinants of ZIKV cell tropism. Our findings suggest that members of the BAF complex are critical for ZIKV infection, and of importance in ZIKV cell tropism and disease.

## 2. Materials and Methods

### 2.1. Viruses, Antibodies, Reagents and Cells

Zika virus (ZIKV; isolate PE243) [30], tick borne encephalitis virus (TBEV; Torö-2003), [31], West Nile virus (WNV; WNV_0304h_ISR00), Japanese encephalitis virus (JEV; Nakayama), yellow fever virus (YFV; Asibi), and dengue virus (DENV serotype-2; PNG/New Guinea C), were kind gifts from S. Vene (Swedish Public Health Agency, Stockholm, Sweden) and propagated as described [32]. Respiratory syncytial virus (RSV-A2-GFP; strain A2) was a kind gift from Dr Mark Peoples, and influenza A virus (IAV-GFP; strain PR8) was a kind gift from Dr Denis Kainov. ZIKV positive cells were stained by immunofluorescense using the pan-flavi antibody 4G2 (Merck Millipore), or with anti-E YFV antibody 2D12 (YFV only). dsRNA intermediates were visualized using the J2 antibody (Scicons). Alexa Fluor-conjugated secondary IgG antibodies were purchased from Thermo Fisher Scientific. The NCI-60 library of cells was obtained from National Cancer Institute. CellTiter-Glo was purchased from Promega; ZIKV primers from Eurofins; QuantiTect primers for GAPDH and γ-actin were purchased from Qiagen, and all HAP1 cells were purchased from Horizon.

### 2.2. NCI-60 Screen for ZIKV Infection

**Figure 1:**
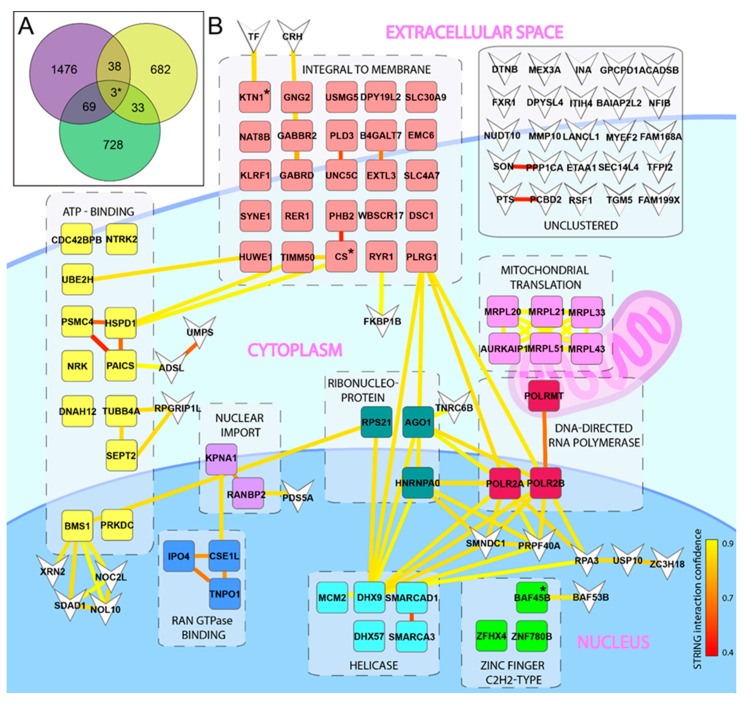
Identification of host factors important for ZIKV infection. (**A**) Venn diagram displaying overlapping genes of importance for ZIKV infection. A library of 60 cell lines (NCI-60) was infected with ZIKV and host factors of importance was identified using the COMPARE algorithm. Overlapping host factors from the NCI-60 (green), the Savidis et al. [28] screen (purple), and the Scaturro et al. [29] screen (yellow) are shown. The three genes identified in all three screens are marked with *. (**B**) Visualization of overlapping host factors that share functional annotations as identified by DAVID are clustered in boxes surrounded by dashed lines at their appropriated cellular localization with simplified annotations. Individual genes present in several functional clusters were allocated to the cluster with the highest enrichment. Yellow/red lines between genes indicate STRING interactions with confidence scoring as indicated by the lower right bar. Genes that were not allocated to a functional annotation cluster are displayed as inverted arrow heads. The three genes identified in all three screens are marked with “*”.

All NCI-60 cells were grown in RPMI-1640 (Invitrogen) supplemented with 10% fetal calf serum (FCS, Invitrogen) and PenStrep (20 U/mL penicillin, 20 µg/mL streptomycin, Invitrogen) according to the NCI-60 recommendation. To ensure high reproducibility and avoid passage-effects, cells were used until reaching the third passage after revival (after which they were discarded). The amount of virus used in the screen was based on a titration in A549 cells aiming for 50% cell death at four days post infection (p.i.). Five cell lines could not be revived and thus the screen was performed with 55 cell lines (Figure 1A). Twenty-four hours after seeding, the cells were infected, or mock treated, using ZIKV (PE243) at/of a multiplicity of infection (MOI) ~0.05 infectious viruses/cell in growth media. At four days p.i., a cell viability assay was performed using CellTiter-Glo (CTG, Promega) according to the manufacturer’s instructions. The ZIKV induced cell death was then obtained by dividing CTG (luminescence) value from the infected cells with the CTG value of uninfected cells followed by subtracting this value (live cells) from 100%. This value was used as a proxy for infection. The average infection values for each cell line were uploaded on NCI-60 website for a COMPARE analysis (https://dtp.cancer.gov/databases_tools/compare.htm (accessed on 26 February 2021)). Data from at least three independent infection experiments were used for the analysis.

Bioinformatic analysis of the three screens resulting in the Venn diagram was generated using the on-line software Venny 2.0 (https://bioinfogp.cnb.csic.es/tools/venny/htm (accessed on 20 November 2020)) using only the genes showing a positive correlation with ZIKV infection in the NCI-60 screen. The Venn diagram was completed with all 756 guideRNA (gRNA)/genes identified in the CRISPR screen [28], and the 1586 targets identified for “capsid” and “E” (Env) [29]. All potential targets listed from each screen were included, independent of the respective cut-off value used in the original publication.

### 2.3. Combined DAVID Functional Annotation Clustering & STRING Interactions

Functional annotation clustering was performed on the 105 overlapping genes from our NCI-60 screen and the screens by [28,29] (Table S3) or on the genes from our NCI-60 screen alone (Table S1), using DAVID Bioinformatics Resources 6.8 [33,34] (http://david.abcc.ncifcrf.govhtm (accessed on 1 October 2016)) with classification stringency set to high. All standard annotation categories were included, and this yielded a total of 1994 genes divided into 77 functional annotation clusters for the NCI-60 hit list (Table S2), and 105 genes divided into 12 clusters for the overlapping list (Table S4).

Functional interactions in these two gene lists were then identified using the STRING database (v.11, https://string-db.org/htm (accessed on 12 August 2021)) [35]. These STRING interactions were combined with the DAVID functional annotation clusters and visualized in a network using Matlab and Cytoscape as described previously [36]. Minor modifications were made to the Matlab code to enable usage in Matlab (R2020a), with visualization in Cytoscape v2.8.1. Individual genes found in multiple clusters were allocated to the functional cluster with the highest enrichment. Genes not belonging to a cluster were visualized as inverted arrowheads. STRING interactions were displayed as solid lines with the colouring (yellow to red gradient) displaying the interaction confidence. A schematic view of a cell with the functional annotation clusters manually put at their approximate cellular localization was created to ease visualisation. For the NCI-60 gene list, the 20 top clusters were chosen. Non-clustered genes were removed due to space limitations.

### 2.4. Quantification of ZIKV Infection in HAP1-WT/HAP1-KO Cells by Immunofluorescence

All HAP1 cells were grown in Iscove’s Modified Dulbecco’s Medium (IMDM, Invitrogen) supplemented with 10% FCS + PenStrep, according to the recommendations by Horizon. For infection, 30,000 cells were seeded into each well of a 96 well plate and infected with ZIKV at a MOI of 0.05. Forty-eight hours post infection, the cells were fixed and stained for ZIKV E protein using 4G2 antibodies. Cells were fixed using 4% formaldehyde for 30 min, washed in PBS and then permeabilized in PBS + 0.5% Triton X-100 + 20 mM glycine for 10 min, washed again in PBS, followed by one-hour incubation with primary antibody, diluted 1:500 in PBS + 10% FCS + 0.05% Tween 80 (Sigma). Cells were then washed 3 times in PBS + 0.05% Tween 80 and stained for 1 h using goat anti-mouse Alexa Fluor 647 (A21236, Thermo Fisher) diluted 1:500. Fluorescence was quantified using a TROPHOS Plate RUNNER HD^®^ (Dioscure, Marseille, France). For every well, the “global fluorescence” was analyzed, and the signal was presented as percentage of the average signal generated with HAP1-WT cells. No background subtraction was necessary as the exposure was set to avoid any background. For every experiment, Hoechst 3322 stain was used to ensure a confluent monolayer. In experiments where the J2 antibody against dsRNA was used, the same staining procedure was performed, but the imaging was performed using a Nikon confocal microscope.

### 2.5. Quantification of ZIKV Infection by qRT-PCR

A total of 100,000 HAP1 cells were seeded into a 24 well plate. After 24 h, the cells were infected with ZIKV at a MOI of 0.01. One hour post infection, the cells were washed, and fresh growth media was added. At 48 h post infection, RNA was collected by the addition of lysis buffer. Total RNA was isolated by an RNA extraction kit from Macherey-Nagel. Gene expression analysis was performed using a One-Step qRT-PCR kit detecting amplification by SYBR green (qPCRBIO). Analysis of viral replication using qRT-PCR was performed using primers for the E gene (Fw: AAGTTTGCATGCTCCAAGAAAAT, Rev: CAGCATTATCCGGTACTCCAGAT). Total RNA was not quantified before the qRT-PCR analysis. For normalization, the Qiagen QuantiTect primers for the gene GAPDH were used. Fold change was calculated by comparison with uninfected control cells harvested at the same time. Expression levels were always normalized to endogenous GAPDH expression using the ∆∆CT method.

### 2.6. Infection of HAP1 Cells by Flavivirus, RSV, and IAV

A total of 30,000 HAP1-WT or BAF45b-KO HAP1 cells were infected by either ZIKV (PE243), TBEV (Torö-2003), WNV (WNV_0304h_ISR00), JEV (Nakayama), YFV (Asibi), or DENV serotype-2 (PNG/New Guinea C) at MOI of 0.05. At 72 h post infection, cells were fixed using 4% formaldehyde for 30 min and stained as previously described. For staining, mouse anti-flavivirus E HB112 ATCC was used against ZIKV, TBEV, WNV, JEV, and DENV; for YFV mouse monoclonal to YFV E CRC 1689 ATCC was used. Both antibodies were diluted 1:1000. The signal was presented as a percentage of the average signal obtained using control (HAP1-WT) cells. No background subtraction was necessary as the exposure was set to avoid any background. For every experiment, Hoechst 3322 stain was used to ensure a confluent monolayer.

In the RSV and IAV infection experiments, infection was carried out in a similar manner to the flavivirus experiments. A total of 30,000 HAP1-WT or BAF45b-KO cells were seeded into a black 96 well plate as previously described. After 24 h, cells were infected with RSV-A2-GFP, or IAV-GFP, for 60 min at 37 °C, using an amount of virus determined from an initial titration in HAP1-WT cells to generate 30–40% infected cells. Infection was allowed to progress for 48 h before fixation using 4% PFA, and imaging of global GFP signal was quantified using a TROPHOS Plate RUNNER HD^®^ (Dioscure, Marseille, France). To avoid fluctuations due to virus-induced cell death, the amount of virus added was titrated to generate approximately 50% infection. In addition, for every experiment, a Hoechst DNA stain was performed to ensure a confluent monolayer at the endpoint. No background subtraction was necessary as the exposure was set to avoid any background.

### 2.7. Statistical Analysis

All experiments were performed at least two times in duplicates, or triplicates. All values are compared to the average GFP signal of the control cells. The results are expressed as means ± standard deviation (SD), and two-way analysis of variance (ANOVA), or a two-tailed Student’s *t*-test, was performed using GraphPad Prism, version 7.00 for Windows (GraphPad Software, San Diego, CA, USA). *p* values < 0.05 were considered statistically significant.

## 3. Results and Discussion

**Figure 2:**
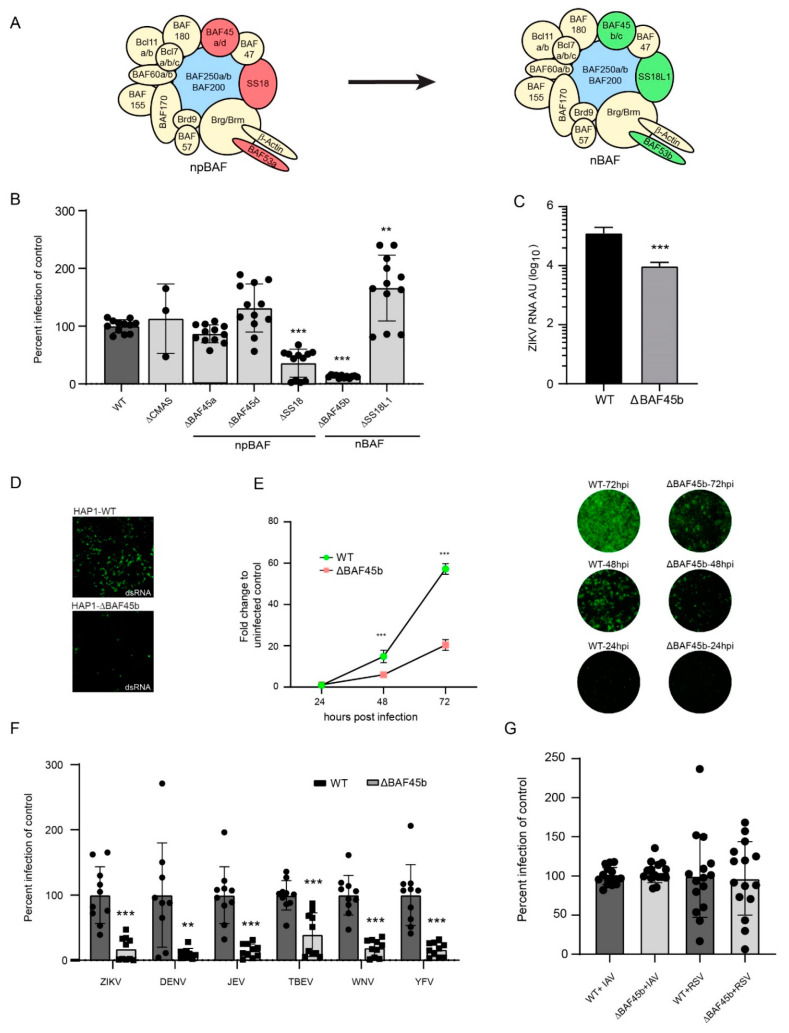
BAF45b and other members of the BAF complexes are required for efficient infection with ZIKV and other flaviviruses. (**A**) An illustration of the BAF complex and how selected subunits are exchanged during transition from neural progenitor BAF to neuronal (postmitotic) BAF. (**B**) Quantification of ZIKV Env protein in WT and HAP1 cells deleted in genes encoding CMAS (control) and members of the BAF complex using pan-flavivirus anti-Env-binding 4G2 antibody 48 h post infection. (**C**) Quantification of ZIKV E RNA in WT and ΔBAF45b HAP1 cells by qRT-PCR 48 h post infection, presented as arbitrary units. (**D**) Staining of WT and ΔBAF45b HAP1 cells using the anti-dsRNA binding J2 antibody at 48 h post infection. (**E**) Quantification of ZIKV Env RNA (left) with representative IF images for each time point (right) in WT and ΔBAF45b HAP1 cells. The pan-flavivirus anti-Env-binding 4G2 antibody was used for ZIKV staining at 24, 48, and 72 h post infection. (**F**) Quantification of flavivirus Env protein in WT and ΔBAF45b HAP1 cells using the pan-flavivirus anti-Env-binding 4G2 antibody 72 h post infection. ZIKV: Zika virus; DENV: dengue virus; JEV: Japanese encephalitis virus; TBEV: tick borne encephalitis virus; WNV: West Nile virus; YFV: yellow fever virus. (**G**) Quantification of GFP—encoded by IAV and RSV—expression in WT and ΔBAF45 HAP1 cells 48 h post infection. All data presented are from two independent experiments, except for (**B**,**E**) (three experiments) and (**G**) (four experiments). Expression levels determined by qRT-PCR were normalized to the endogenous GAPDH expression and calculated using the ∆∆CT method. Data is plotted as average with standard deviation (SD) and compared to the average of the control. In (**B**) statistical significance was determined using a two-way ANOVA *** *p* > 0.001. In (**C**,**E**,**F**) statistical significance was determined by using a student’s t-test where ** *p* > 0.01 and *** *p* > 0.001.

To identify novel host factors of importance for ZIKV infection, we took advantage of the NCI-60 library of 60 human cell lines that have been mapped by mRNA micro array [37], and successfully used to identify the mechanism(s) of action of several drugs used in cancer therapy [38]. This tool has also been used for identification of PDGFR as a cellular receptor for adeno-associated virus [39]. The cell lines represent several different tissues and organs, including the CNS. We used this library to quantify total cell death as a consequence of released infectious progeny virus, in order to identify host factors involved at any step in the entire ZIKV life cycle. Firstly, upon examining the infection profile, we found no obvious preference for cell types of any given origin, or, for cell types growing faster or slower (Figure S1). The infection profile was compared to the available mRNA expression values of all the investigated cell lines using the NCI-60 COMPARE algorithm, and, as expected, we identified genes with positive, or negative, correlation to infection (Table S1). Initial functional clustering and interaction analysis of these genes did not reveal any evident targets for further validation (Figure S2, Table S2). To narrow down the hit list, we decided to search for overlapping hits in our list of genes with two already published screens: a genome wide CRISPR screen [28] and an overexpression-pulldown proteomics screen [29]. For comparison, we selected the 833 genes with a positive correlation to infection from our NCI-60 gene list; the whole list of 756 detected gRNA/genes from the CRISPR screen [28]; and the 1586 genes identified via overexpression-pulldown using the envelope (E) and capsid (C) proteins [29]. For an unbiased approach, and to allow for identification of novel targets with otherwise low hit scoring, we included all genes from these two screens independent of any thresholds used in the original publications. Overlapping genes from the three different screens were visualized by a Venn diagram and resulted in a total list of 105 genes that were present in our NCI-60 hit-list, and in at least one of the other screens (Figure 1A, Table S3). Functional annotation clustering combined with functional interactions of these 105 genes points out twelve functional clusters (Table S4) in total. The helicase and zinc finger C2H2-type clusters contain genes including SMARCAD1, SMARCA3, and BAF45b, which are associated with transcription and epigenetic regulation (Figure 1B). In our screen (but not in the other screens), we also observed correlations with additional genes related to these clusters: positive correlation was observed with SMARCA1, and negative correlation with BAF45D, SMARCA2 (BRM), SMARCA4 (BRG), and SS18 (Table S1). Strikingly, several of these genes encode protein subunits of the BAF transcription complex (Figure 2A). One of these genes (BAF45b, also known as DPF1) was identified in all three screens (Figure 1A, Table S3), and encodes a protein important in neuronal differentiation [40].

BAF45b is largely expressed in the brain (neurons) and in testis (haploid spermatids) [41,42], which correlates with the tropism of ZIKV infection [43]. BAF45b is also expressed in high amounts in the near haploid HAP1 cell line [41,42], derived from the chronic myeloid KBM-7 cancer cell line [44]. Using HAP1 cells as a model to understand the role of the BAF complex in cancer, it was concluded that different BAF subcomplexes play a role in genome-wide chromatin organization [45]. Thus, we selected WT HAP1 cells and cells with BAF45b expression knocked out (KO; ΔBAF45b), to investigate a possible role of BAF45b in ZIKV infection. Upon visual inspections, there was no obvious difference in growth kinetics and/or morphology of ΔBAF45b, or any other KO, compared to the parental HAP1 cells, thus suggesting that the absence of BAF-KO did not affect the viability of the cells. Quantification of cells stained for anti-ZIKV Env antibodies at 48 h post infection revealed that BAF45b expression was important for ZIKV infection as only residual (7.5%) infection was observed in ΔBAF45b cells, as compared to infection in WT cells and in HAP1 cells with CMAS expression knocked out (ΔCMAS; CMAS is involved in biosynthesis of sialic acid monosaccharides and used here as a control) (Figure 2B). We also quantified Env expression in additional, commercially available HAP1 cells knocked out for other members of the BAF complex. ZIKV infection was not affected in cells lacking expression of BAF45a or BAF45d as compared to control HAP1 cells, but at least one additional member of the BAF complex, SS18, was needed for efficient ZIKV infection, as infection was reduced in these cells by 75% (Figure 2B) compared to control HAP1 cells. We also noted a slight increase in infection in SS18L1 KO cells. Whereas BAF45b is only present in the complex found in postmitotic neurons (nBAF), SS18 is only detected in the complex found in neural progenitor cells (npBAF). Since, in the context of CZS, ZIKV prefers to infect NPCs over post-mitotic neurons [8], it is striking that BAF45b—a marker for post-mitotic neurons and a member of the nBAF complex—was important for ZIKV infection. Given the pronounced tropism for neural progenitor cells, it was expected that ZIKV infection was also reduced in HAP1 cells deficient in SS18, both being markers for NPCs and members of the npBAF complex. As the other subunits that are switched during differentiation (BAF53a, BAF45c, and BAF53b) are not expressed in HAP1 cells, corresponding cells are not available, and we could therefore not investigate their effect in this model system. Nevertheless, these results suggested a role of the BAF complex subunits in ZIKV infection. This was confirmed as we also observed a reduced production of ZIKV Env RNA by qRT-PCR (Figure 2C) and dsRNA (an intermediate product produced during ZIKV replication) by immunostaining (Figure 2D) in ΔBAF45b cells as compared to WT cells (both 2C and 2D) at 48 h post infection. Furthermore, in a time-course experiment we observed an increased infection (staining of Env protein) also in ΔBAF45b cells, suggesting that BAF45b is not essential for infection, but is, however, important, as infection increased substantially more over time in WT cells (Figure 2E).

Next, we investigated if BAF45b was important only for ZIKV, or also for infection of other related and unrelated viruses. Strikingly, all investigated flaviviruses infected ΔBAF45b cells less efficiently than WT cells at 72 h post infection (Figure 2F). We did not observe any particular correlation between viruses with more (ZIKV; Japanese encephalitis virus: JEV; tick-borne encephalitis virus: TBEV; West Nile virus (WNV)) or less (dengue virus: DENV; yellow fever virus: YFV) neuronal tropism, suggesting that the subunits are not directly involved in neuronal tropism, but rather are more important generally in flavivirus infections. Two other unrelated viruses (influenza A virus; IAV, and respiratory syncytial virus: RSV, of the *Orthomyxoviridae* and *Paramyxoviridae* families) infected ΔBAF45b cells with similar efficiency as WT cells, as quantified by virus-encoded GFP expression (Figure 2G) at 48 h post infection. This suggests that BAF45b is important for infection by arthropod-borne flaviviruses with positive sense, single stranded RNA, but not by IAV or RSV with negative sense, single-stranded RNA. It remains to be investigated if BAF45b and other subunits of the BAF complex are important for infection of other non-flavivirus members of the Flaviviridae family, as well as viruses of other families, presumably Baltimore class IV viruses with positive sense, single-stranded RNA.

Our results do not allow us to conclude about specific mechanisms, but as the BAF complexes are involved in transcription and epigenetic regulation of genes controlling neural development, it is plausible that subunits of the BAF complexes–and/or the genes controlled by these complexes—are involved in flavivirus infection, and thus, also in flavivirus pathogenesis. This is supported by data showing (i) that BAF53a is repressed by sequences in the 3′ untranslated region corresponding to the recognition sites for miR-9* and miR-124, which are selectively expressed in post-mitotic neurons [18] and known to be modulated during flavivirus infection [46], and, (ii) by previous findings showing that upregulation of miR-9 is associated with ZIKV infection in mice and microcephaly [13]. Thus, our data provide a hitherto unrecognized link between subunits of the BAF complexes and flavivirus infection, which is potentially associated with CZS. Additional support for an important role of BAF complex subunits in flavivirus replication is provided by the identification of BAF45b and additional members of the BAF complex, in other published screens [30,47,48,49,50]. Given the known function of the BAF complex and other, similar epigenetic regulators of transcription, the results presented in this study suggest that regulators of epigenetic transcription may be important determinants of virus infection, tropism, and disease, and constitute potential targets for antiviral treatment.

## Supplementary Materials

The following are available online at https://www.mdpi.com/article/10.3390/v13102007/s1, Figure S1: Summary of infection data from the NCI-60 screen, Figure S2: Visualization of host factors identified in the NCI-60 screen, Table S1: NCI-60 screen—Results of the COMPARE analysis, Table S2: NCI-60-screening data—Functional clustering of genes in A using DAVID functional annotation clustering, Table S3: Overlapping genes—List of 105 overlapping genes found in the NCI60 screen and in one, or both of the screens carried out previously [28,29], Table S4: Overlapping genes—Functional clusters of the genes in A using DAVID functional annotation clustering.
