# Supplementary material for: BAF45b Is Required for Efficient Zika Virus Infection of HAP1 Cells
Source: Viruses. 2021 Oct 6;13(10):2007. doi: 10.3390/v13102007 (PMC8540262; doi:10.3390/v13102007)
Supplement: Supplementary file 1 [file viruses-13-02007-s001.zip › SUPPLEMENTAL MATERIAL CLEAN.pdf]

SUPPLEMENTAL MATERIAL

Supplemental figure S1, S2

Supplemental table S1, S2

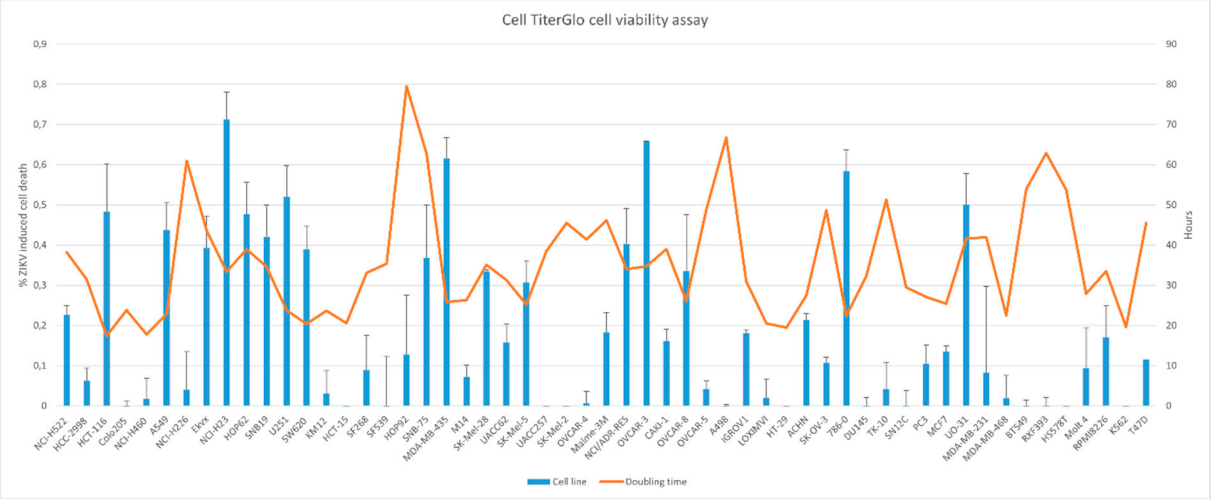

Figure S1. Summary of cell death data from the NCI-60 screen. Zika virus infection is shown as blue bar graphs representing % Zika virus induced cell death compared to an uninfected control. The “infection value” was obtained by dividing CTG (luminescence; corresponding to cell death) value from the infected cells with the CTG value of uninfected and subtracting this percentage from 100%. The cell doubling time for each cell line is represented by the orange line (doubling time obtained from NCI). If the experiment-to-experiment deviation was high after three individual experiments two additional experiments were performed. Data is shown as mean ± standard deviation.

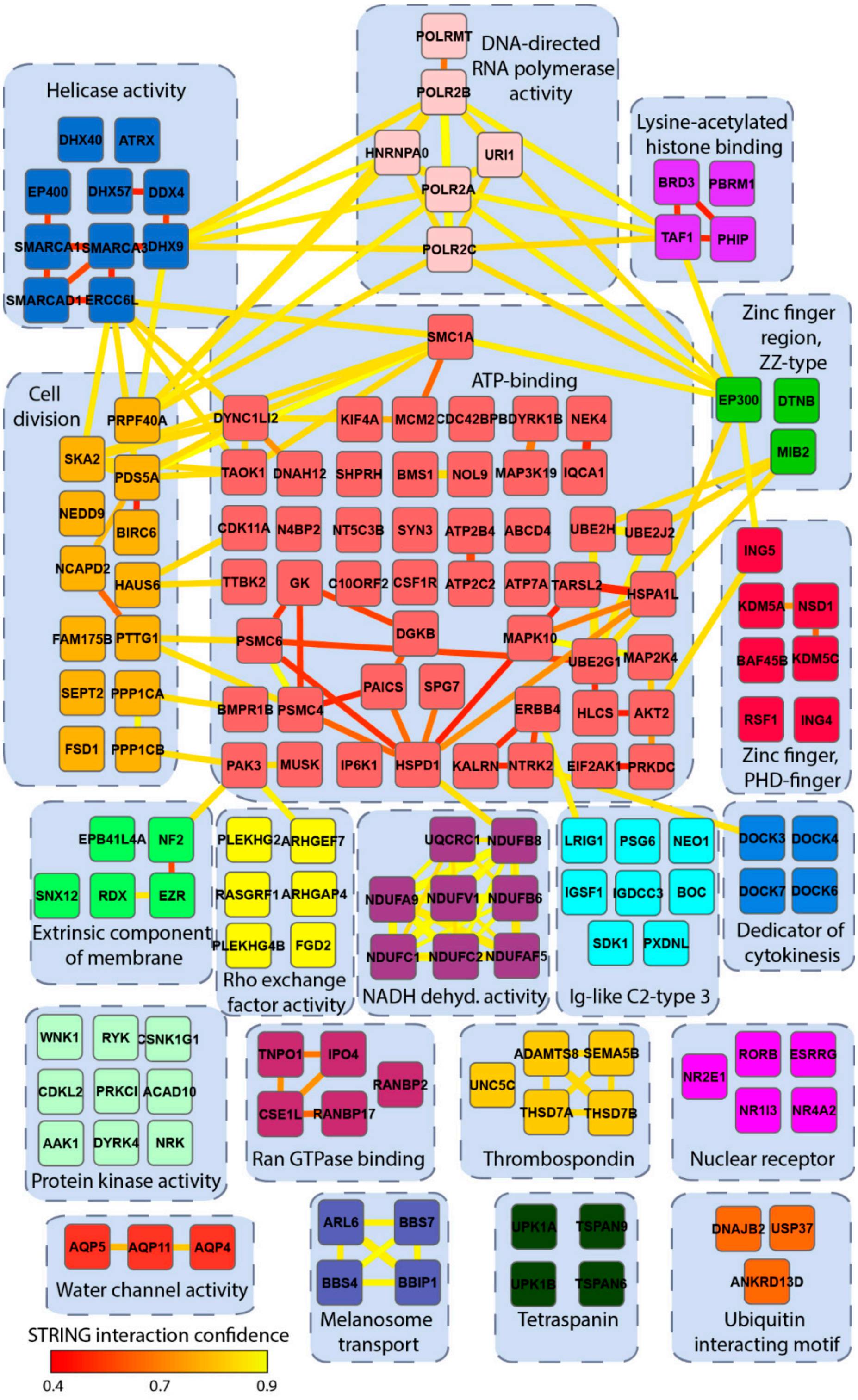

Figure S2. Visualization of host factors identified in the NCI-60 screen. The top 20 functional annotation clusters identified with DAVID are displayed in boxes with dashed lines along with their interactions identified by STRING. Individual genes present in several functional clusters were allocated to the cluster with the highest enrichment. Yellow/red lines between genes indicate STRING interactions with confidence scoring as indicated by the lower left bar.

Supplemental tables

Supplemental table S1. NCI-60 screening data. A) Results of the COMPARE analysis, containing genes with both positive and negative correlation to ZIKV infection. B) Functional clustering of the genes in Table S1A using DAVID functional annotation clustering.

Supplemental table S2. Overlapping genes. A) List of 105 overlapping genes found in our NCI-60 screen and in one, or both, of the screens carried out previously (Scaturro et al., 2018; Savidis et al., 2016). B) Functional clustering of the genes in Table S2A using DAVID functional annotation clustering. The Y sign in the table means presence in the mentioned screen.
